# Maintaining Places of Social Inclusion: Ebola and the Emergency Department

**DOI:** 10.1177/0001839220916401

**Published:** 2020-03-20

**Authors:** April L. Wright, Alan D. Meyer, Trish Reay, Jonathan Staggs

**Affiliations:** 1 University of Queensland; 2 University of Oregon; 3 University of Alberta; 4 Christian Heritage College

**Keywords:** place, institutional work, institutional maintenance, institutional disruption, qualitative research, process research, social inclusion, health care, professions, custodianship, pandemic, coronavirus

## Abstract

We introduce the concept of places of social inclusion—institutions endowed by a society or a community with material resources, meaning, and values at geographic sites where citizens can access services for specific needs—as taken-for-granted, essential, and inherently precarious. Based on our study of an emergency department that was disrupted by the threat of the Ebola virus in 2014, we develop a process model to explain how a place of social inclusion can be maintained by custodians. We show how these custodians—in our fieldsite, doctors and nurses—experience and engage in institutional work to manage different levels of tension between the value of inclusion and the reality of finite resources, as well as tension between inclusion and the desire for safety. We also demonstrate how the interplay of custodians’ emotions is integral to maintaining the place of social inclusion. The primary contribution of our study is to shine light on places of social inclusion as important institutions in democratic society. We also reveal the theoretical and practical importance of places as institutions, deepen understanding of custodians and custodianship as a form of institutional work, and offer new insight into the dynamic processes that connect emotions and institutional work.

In most Western societies, the democratic state establishes and funds particular places that play a special role in meeting citizens’ needs and that contribute to the functioning of societies and communities. For example, citizens can freely access public emergency departments for medical care, public schools for education, public libraries and museums for information and culture, public law courts for justice, and public parks and community centers for recreation and community belonging. The open accessibility of these places is a hallmark of the values of the democratic state and its commitment to the welfare of its citizens (Parkinson, 2012).

Several literatures help us understand the characteristics and functioning of these special places. Scholars of humanistic geography and sociology have established the concept of place broadly as a combination of geographic location, materiality, and meaning (Tuan, 1977; Gieryn, 2000). Building on this literature, Carr et al. (1992) focused specifically on the importance of public places—those designed and built to provide universal accessibility for all citizens to essential human services—and proposed that such places are where citizens go to make “claims” for access to services. The literature on institutional theory in organizational studies, in contrast, has so far engaged only minimally with the concept of place, primarily considering place as merely the research setting (Lawrence and Dover, 2015). Yet because an institutional approach inherently draws attention to the role of institutions in bringing order, stability, and meaning to society (Scott, 2014), there are obvious synergies in combining aspects of institutional theory with the concept of place.

Taken together, these theoretical perspectives lay out the importance of a special type of place whose functioning as an institution is integral to democratic society because it fulfills normative social purposes (Francis et al., 2012; Parkinson, 2012). However, this type of taken-for-granted place has not been explicitly identified or conceptualized in prior research. We label this institution a “place of social inclusion.” Building on the currently disparate approaches, and through our analysis of an empirical case, we develop the definition of a place of social inclusion: an institution endowed by a society or a community with material resources, meaning, and values at geographic sites where citizens have the right to access services for specific needs.

In developing our concept of a place of social inclusion as an important institution of the democratic state, we focus attention on how the combination of place and institution produces precariousness. Early institutional scholars cautioned that institutions’ values are inherently precarious because their existence at the macro level of society depends on organizations and individuals reproducing them at the micro level in actions and interactions (Selznick, 1957, 1992). In considering a place of social inclusion as an institution, we draw on the place literature to suggest that the institution’s precariousness is inherent in the local character of places and place claiming (Carr et al., 1992; Gieryn, 2000). By this we mean the societal-level value of providing universal accessibility to essential services for all citizens is precarious because it must be continuously accomplished at the local level each time a citizen makes a claim for services from a specific geographically bounded site in a neighborhood, town, or city. At the local level, the accessibility of any particular site that is a place of social inclusion—for example, a local emergency department or public school—may be affected by factors such as population growth, income inequalities, natural disasters, and social ills like poverty, illiteracy, homelessness, and poor physical and mental health. Gun shootings occur in local schools, terrorists attack citizens in public open places, crime and violence assail neighborhoods, and pandemics and infectious diseases spread across national borders and within local communities, as the 2020 pandemic of coronavirus disease (COVID-19) showed while we were finalizing this research. We posit that places of social inclusion are constituted as universally accessible institutions of the democratic state, yet the perpetuation of these institutions is consistently challenged by such ongoing threats. How, then, are places of social inclusion maintained?

We investigate this research question through a longitudinal field study of the emergency department of a public hospital in an Australian city, which we argue is a compelling empirical example of a place of social inclusion. The Australian government establishes and supports public emergency departments in local places to offer all citizens access to care and treatment for their acute health needs. By undertaking observations and interviews at our emergency department fieldsite, we were able to examine the everyday struggles involved in accomplishing the societal-level value of social inclusion through universal access to medical care at a local place. The precariousness of the emergency department as a place of social inclusion was cast into bold relief when our fieldwork was punctuated by the Ebola outbreak in 2014, offering a unique opportunity to explore and understand both the ordinary and extraordinary efforts needed to maintain places of social inclusion as important institutions in democratic society.

## Institutions and Place

Over the past few decades, geographers and sociologists have examined the human experience of place. Humanistic geographers conceive of places as geographic sites that become meaningful through people’s social interactions and emotional attachments (Relph, 1976; Tuan, 1977). Sociologists have focused on how insiders and outsiders shape the meaning and character of a place (Lewicka, 2011). These literatures point to specific places—such as buildings, neighborhoods, cities, towns, and other public and private sites—as “centers of value and significance” (Tuan, 1977: 415).

Researchers distinguish three essential elements of a place (Agnew, 1987; Gieryn, 2000). First, a place is a geographic location, “a unique spot in the universe” distinct from neighboring places (Gieryn, 2000: 464). Second, a place has physical form that includes natural and built resources, material objects, and organizing routines (Stedman, 2003). Third, people invest a place with special meaning and value based on their relationship with its history and identity (Brown-Saracino, 2017). A place comes into existence and endures when people “recognize themselves and others as part of a common enterprise with mutual meanings and experiences” (Kaufman and Kaliner, 2011: 122).

People’s capacity to associate a place with a common enterprise and ascribe enduring meanings suggests that places are institutions. Scott (2014: 56) defined institutions as comprising “regulative, normative and cultural-cognitive elements that, together with associated activities and resources, provide stability and meaning to social life.” While institutional scholars have rarely postulated meaningful relationships between places and institutions (see Lawrence and Dover, 2015, for an exception), Scott’s (2014) definition implies that a place should be conceptualized as an institution. A place is regulated by laws, rules, and codes of conduct that seek to control and order how people interact with and in that place. A place is supported normatively by values and beliefs that define what inhabitants should strive to attain and how. Finally, a place will evoke shared meanings and “a way of seeing, knowing and understanding the world” that is cognitively and affectively accepted by people who inhabit that place (Cresswell, 2014: 11). Moreover, because institutions are multi-level systems, both a higher-order place like the world system and a local place like a county fair can be institutions (Barley, 2008). The world is “filled with significant places” (Relph, 1976: 1) that are institutions and that are intrinsic to the constitution and reproduction of social life (Giddens, 1984). In this paper, we draw attention to the institution of a place of social inclusion.

### Places of Social Inclusion

Building on the previous literature about place and aspects of institutional theory, we conceptualize a place of social inclusion as a distinctive type of institution commonly associated with the democratic state. This institution is instantiated in local geographic sites that are publicly accessible to citizens with specified human needs and are infused with meaning and values associated with normative social purpose. Established theory about public places focuses on the importance of being situated locally in neighborhoods, towns, cities, states, and nations (Francis et al., 2012; Parkinson, 2012). Because places are geographically bounded by their natural and physical location and the buildings and material objects assembled there (Gieryn, 2000), the physicality of places affects the way in which they are resourced to accomplish normative social purposes. Although previous literature on public places has not stressed the characteristic of social inclusiveness, to varying degrees it is inherent to discussions of public access to places such as emergency departments, public schools, libraries, recreational parks, welfare offices, community centers, and courts of law.

Insights from the literature suggest the nature of human needs and the depth of association with, and accomplishment of, values of social inclusion involving equality, dignity, and human rights vary across different public places. Some places are explicitly created and maintained to accomplish social inclusion for groups marginalized with respect to race, disability, or other characteristics (Bolt, Burgers, and van Kempen, 1998; Hall, 2010; Brown-Saracino, 2017). Other places fulfill a social purpose that is sometimes associated with universal access to basic human services (Altman and Zube, 1989; Carr et al., 1992). These studies of public places provide a foundation for considering how some particular places can be established and maintained as places of social inclusion.

Combining the above ideas with concepts from institutional theory conceptualizing institutions as multi-level phenomena (Barley, 2008), we posit that a place of social inclusion is nested across societal and local levels. The societal level captures the regulative and cultural–cognitive elements of a place of social inclusion in the democratic state and the normative values it is expected to accomplish for citizens through meeting specific needs. Depending on the precise nature of these needs, places of social inclusion are embedded in the state and also intersect with other higher-order institutions in society such as the medical, teaching, and legal professions. The local level reflects the specificity of the geographic places in which the institutional values of a place of social inclusion are accomplished. That is, human needs are met at local sites—such as a local emergency department or public school—that are resourced by the state to actualize the institutional value of social inclusion. Because places at the local level are “forever precarious and contested” when they nest within societal-level systems (Gieryn, 2000: 472), a place of social institution is an inherently precarious type of institution.

### Maintaining Places of Social Inclusion

Two literature streams offer preliminary guidance on how places of social inclusion might be maintained. Geographers and sociologists pursue one stream, focusing on how people interact with a place (Relph, 1976; Lewicka, 2011). This literature posits a reciprocal relationship between people and place (Kaufman and Kaliner, 2011). Places are created, reproduced, and transformed by the human activities and social relations that transpire in a particular locale, as people experience and interpret the place’s historically contingent meanings, values, routines, and resources (Tuan, 1977; Agnew, 1987). Cognitions and emotions shape how people interact with each other and with the material aspects of place (Scannell and Gifford, 2010). This literature conceptualizes a person who visits a public place to access its material and symbolic resources as “making a claim” on the place (Carr et al., 1992). The staff of the public place must respond by “strik[ing] the right balance among various claims on its use and meaning” (Carr et al., 1992: 20) and ensuring “place claims are . . . being noticed and taken seriously” (Parkinson, 2012: 1). However, the everyday cycle of claim-making and responding is not well understood (Patterson and Williams, 2005; Molotch, 2011). Researchers know little about the behavioral routines that maintain the rhythm of life of a place and how they shape people’s experiences of material resources and their enduring sense of place (Stedman, 2003).

Other clues about how places of social inclusion might be maintained can be found in the literature on institutional work. Lawrence and Suddaby (2006: 230) defined the work of maintaining institutions as “supporting, repairing, or recreating the social mechanisms that ensure compliance.” Implicit in most studies is an assumption that place is just the site where actors perform institutional work directed at other institutions. Researchers have highlighted how the Cambridge University dining hall (Dacin, Munir, and Tracey, 2010) and English County Cricket grounds (Wright and Zammuto, 2013) offer settings where actors perform institutional work that (re)produces institutions of social class. Nazi concentration camps have been viewed as settings where institutional work creating social oppression was undertaken (Marti and Fernandez, 2013). Institutional work maintaining professional occupations goes on in museums, law courts, restaurants, and hospitals through the everyday actions of curators (Blagoev, Felten, and Kahn, 2018), lawyers and advocates (McPherson and Sauder, 2013; Siebert, Wilson, and Hamilton, 2017), chefs (Gill and Burrow, 2018), and physicians and nurses (Reay, Golden-Biddle, and GermAnn, 2006; Kellogg, 2009; Wright, Zammuto, and Liesch, 2017). Place was not the focus in the aforementioned studies, but Lawrence and Dover (2015) have drawn attention to how place influences institutional work, showing how places serve as “social enclosures” that contain, “signifiers” that mediate, and “practical objects” that complicate institutional work. We draw inferences from the established literature to suggest three tentative insights into how the institutional work associated with maintaining a place of social inclusion might play out.

The first insight concerns the institutional work of the actors who respond to claims on a place of social inclusion. Selznick (1957: 94) argued that societal values are protected by institutional “guardians”: professionals working inside formal organizations who are entrusted with institutional values and given autonomy to defend them from subversion by other goals. While some scholars have applied Selznick’s concept of guardians (Kraatz, Ventresca, and Deng, 2010; Kraatz and Flores, 2015), others have invoked Soares’ (1997) term “custodian” to designate the caretakers of values, traditions, and institutionalized practices (Dacin, Dacin, and Kent, 2019). For Soares (1997: 14–15), custodians are “practitioners who have a sense of community . . . [and] a sense of custodianship for the tradition’s present and future prospects.” Howard-Grenville and colleagues (2013: 119) used the term “custodians” to label community members who “actively and tenaciously conserved and protected the [place] identity” of TrackTown USA. Taken together, these studies suggest that places of social inclusion may be maintained by custodians.

The second insight sheds light on the processes and emotions that might be involved in custodians’ institutional work. People are motivated to engage in maintenance work when they have cognitive and emotional investment in an institution (Voronov and Vince, 2012). While the institutional literature on emotions is still underdeveloped, studies have suggested that emotions like shame and fear of punishment discourage deviations from prescribed ways of thinking, acting, and feeling (Creed et al., 2014; Gill and Burrow, 2018). Other studies have shown that moral emotions—which the psychology literature links to the interests and welfare of society (Haidt, 2003)—motivate reflective action to maintain, protect, and defend institutional values and practices (Fan and Zietsma, 2017; Toubiana and Zietsma, 2017; Wright, Zammuto, and Liesch, 2017). This emerging body of research suggests that custodians may be cognitively and affectively motivated to undertake maintenance work to protect the values of a place of social inclusion.

The third insight concerns the intentionality of the work involved in maintaining a place of social inclusion. Actions associated with maintenance may not always be obvious because the custodians who inhabit institutions are engaged in the usual day-to-day affairs of their workplace (Hallett and Ventresca, 2006; Fine and Hallett, 2014). Maintenance work may be “nearly invisible and often mundane” (Lawrence, Suddaby, and Leca, 2009: 1) when custodians support an institution by complying with regulations, enacting normative routines, and performing rituals (Lawrence and Suddaby, 2006; Dacin, Munir, and Tracey, 2010). Although these actions are institutionally conditioned, custodians are still able to engage in low-level intentional action by making small-scale incremental choices between sets of institutionalized practices and routines (Battilana and D′Aunno, 2009). However, because breakdowns in institutionalized practices are inevitable, “active and intentional custodial work may be [necessary] for the continued stability of most institutions” (Lok and de Rond, 2013: 187). More intentional maintenance work involves self-conscious action and reflection by custodians (Battilana and D′Aunno, 2009), as well as deliberate efforts to resist change and to defend and repair an institution whose survival is threatened (Kellogg, 2009; Currie et al., 2012).

We bring together insights from existing literature to conceptualize places of social inclusion as important institutions of normative social purpose established by the democratic state that have so far been largely ignored. Identifying and conceptualizing these places is critical because further study holds potential to advance theory about the connections between place and institutions. This is also important because the societal-level value of social inclusion is challenging to accomplish in publicly accessible and geographically bounded sites at the local level. Prior studies have suggested that places of social inclusion may be maintained by custodians, and we need to know more about how such precarious institutions can survive. Seeking to develop a deeper understanding of how the institutional work of custodians can maintain a place of social inclusion, we conducted a longitudinal qualitative study of the emergency department of a public hospital in Australia.

## Methods

### Research Setting

Emergency departments (EDs) are compelling cases of places of social inclusion. In most Western countries, including the United Kingdom, Canada, Australia, and New Zealand, the state funds EDs as “the accessible front door to the healthcare system” (Bailey, Murphy, and Porock, 2011: 1131). In the U.S., the Emergency Medical Treatment and Labor Act requires EDs to evaluate and treat all persons needing emergency medical care regardless of their ability to pay; most Americans regard having a nearby ED as “equally or more important than having a nearby library, public health clinic, fire department, or police department” (Baehr, Martinez, and Carr, 2017: 229). EDs are staffed 24 hours a day to provide urgent medical attention to people in need (Coget and Keller, 2010), and citizens with acute illnesses or injuries go to these universally accessible sites to receive treatment from emergency physicians and nurses (Kellermann, 2006; Wright et. al., 2016). We focused our investigation on an ED in Australia, where the state funds EDs based in public hospitals in metropolitan, regional, and rural locations. Signage of white lettering on a red background, recognizable to all Australians, denotes that a place has official status as an ED and is accessible free of charge. In 2015–2016, over 7.5 million patients visited EDs in Australia (AIHW, 2016).

### Data Collection

This study is part of an ongoing research project focusing on emergency physicians and nurses and their role in Australia’s health care system. Data were collected at the ED of a large public hospital located in the inner city of a major metropolis. Each day, over 200 patients arrived at our ED fieldsite through large glass entry doors that were open 24 hours a day, or they arrived by ambulance. Patients were assigned to a category on the Australasian Triage Scale and streamed by the triage nurse as follows: (1) patients suffering imminently life- and limb-threatening conditions—such as a heart attack, stroke, or major trauma—were streamed to a “Resuscitation Zone” with advanced equipment and beds; (2) patients with urgent, semi-urgent, and non-urgent needs were streamed to an “Acute Zone” of examination bed cubicles with basic equipment; and (3) patients whose condition could be treated rapidly and discharged, such as minor lacerations and fractures, were streamed to a “Fast Track Zone.” About 80 percent of emergency patients were able to be treated and discharged, while the remaining 20 percent were judged to be sufficiently unwell to require hospital admission.

We collected observational, interview, and archival data. Our primary data source was observational fieldnotes taken while shadowing emergency physicians and nurses as they assessed, diagnosed, treated, and discharged or referred patients within the Acute Zone, which was the focus for this study. The Acute Zone responded to the highest volume of patients and experienced the greatest difficulties with patient flow and overcrowding, suggesting that custodian work to maintain the ED as a place of social inclusion was particularly important in this zone. We collected a total of 210 hours of observational data over a six-month period, with observations recorded as handwritten fieldnotes and typed up after each shift. These observational data were supplemented with 47 interviews with senior emergency physicians who oversaw the everyday work of the ED: 15 senior emergency physicians were interviewed twice, and 17 were interviewed on a single occasion. Interviews were semi-structured, with questions designed to elicit accounts of the emergency physician’s work as a custodian of the ED as a place of social inclusion. Interviews lasted between 60 and 90 minutes and were digitally recorded and transcribed. We also interviewed nine residents who were undergoing specialty training in emergency medicine and 29 nurses about their experiences in the ED, with these interviews typically lasting 30 minutes. Four additional interviews, lasting between 60 and 90 minutes, were conducted with hospital executives, and we attended a number of staff meetings, training sessions, and strategic planning days. Archival documents, many of which were publicly available, provided background information on the organizational and professional context.

During our data collection, the outbreak of the Ebola virus in West Africa created a sudden jolt (Meyer, 1982). In August 2014, the World Health Organization (WHO) declared a “public health emergency of international concern.” WHO estimated a possible 20,000 cases from the Ebola outbreak and a mortality rate of 70 percent. Around 10 percent of the cases and fatalities were health care aid workers. The first cases of Ebola transmission outside of Africa occurred in October 2014, when nurses in Spain and the U.S. tested positive for Ebola after treating travelers who had contracted the virus in Africa. During the epidemic, the Australian government designated our fieldsite hospital as one of Australia’s “Ebola response and treatment hospitals.” Although the ED never treated a confirmed case of Ebola, staff undertook preparations as the state’s frontline responders and dealt with several persons suspected of being infected with Ebola. We included probing questions about Ebola in our interviews, engaged in informal conversations and debriefs during observations, and collected archival documents, including WHO updates, government reports, and media releases.

### Data Analysis

Data analysis followed established procedures for inductive theory building from qualitative data (Corbin and Strauss, 2008). We used NVivo 9 and Excel software to assist with coding. Our initial focus was the observational fieldnotes, which captured in real time the day-to-day activities of doctors and nurses responding to persons who presented to the ED. Consistent with the place literature (Carr et al., 1992; Parkinson, 2012), we conceptualized each presentation as a “claim” made on the “place-specific resources” of the fieldsite ED and viewed doctors and nurses as the “custodians” tasked with allocating resources to satisfy claims. As we read our fieldnotes, we were struck by the consistent responses of doctors and nurses regarding their responsibility to ensure the ED remained accessible to all citizens. As part of their approach to providing services, we noticed that as they evaluated cues about each claim, doctors and nurses experienced different levels of tension between the ED being an inclusive place for everyone and having sufficient resources at the local place to respond adequately to all claims.^1^

We observed that doctors and nurses served as custodians of resources through two types of work when responding to a claim. The first type was activated when doctors and nurses experienced a low level of tension between inclusion and resources and interpreted the basis for a person’s claim as an immediate or recurrent health need. Here, we noticed that doctors and nurses tended to feel emotions of low intensity. After consulting the literature (Hoffman, 2000; Haidt, 2003), we categorized these feelings as *moral emotions* because the custodians expressed a desire to “do the right thing” for the patient and for society when allocating resources to the claim. We labeled this custodianship as *resource-rationing work*.

The second type of custodian work was activated when there was a high level of tension between social inclusion and finite resources. Here, we noticed that moral emotions were more intense. Custodians felt deep compassion for patients and families, and they prioritized the highest societal-level ideals of a place of social inclusion. They responded by providing extraordinary access to resources, which we labeled as *resource-enabling work*.

From this first analytical cycle, we speculated that ordinary resource-rationing work and extraordinary resource-enabling work represent micro-processes of custodianship that resolve the inclusion–resource tension, thereby maintaining the ED as a place of social inclusion. To probe our hunch, we turned next to the interview data.

Reading the different transcripts, we noted that doctors and nurses described processes similar to those we observed in the real-time fieldnotes. As we reviewed the interviews, we noticed how doctors and nurses portrayed their experience of, and response to, the inclusion–resource tension as relating to the level of the claim while also recognizing that pressure on resources built up at the level of the ED as a local place. This was most evident in comments about responding to claims during busy shifts and working hard to avoid “going on bypass.” State regulations permit Australian emergency departments experiencing extreme resource pressures to declare a “bypass” situation, temporarily closing the hospital to new patients arriving via ambulance. Our fieldnotes contained one instance when our fieldsite ED declared a bypass. This suggested the ED could be disrupted as a local place of social inclusion when custodians’ efforts to respond to claims through resource-rationing and resource-enabling work failed.

Yet custodianship associated with the inclusion–resource tension only partly explained our data. The jolt from the Ebola crisis gave us the opportunity to investigate a second tension that arises when safety of the local place conflicts with social inclusion. We were struck by the anomaly that while potential Ebola claims evoked unmistakable fear among custodians, other potentially harmful claims did not. Interviewees described how they were “used to” dealing with physical harm from persons who were behaving violently and with the infection risk of “well-known” transmissible diseases. Consulting the literature for guidance, we speculated that Ebola confronted custodians with a decision situation reminiscent of “Knightian uncertainty,” when prediction of future outcomes is impossible. (Knight, 1921). In contrast, claims associated with other threats of harm presented decision situations involving “risk,” when possible future outcomes are known and probabilities can be attached. The literature also offered guidance on the differences between *fear*—a basic emotion that is immediately felt and triggers individual self-defense responses (Ekman, 1992)—and the higher-order and more reflective moral emotions that we had noticed custodians experiencing toward the inclusion–resource tension. We sensed that being able to manage fear by mitigating risk was fundamental to custodian work that maintained the ED as a place of social inclusion in the face of the inclusion–safety tension.

Armed with these distinctions, we returned to our data for a second cycle of more refined coding. Our initial interest lay in elaborating custodian work for claims involving the inclusion–resource tension. Reviewing our fieldnotes and applying Trefalt’s (2013: 1807) method of viewing every claim as an “episode” that constituted a unit for data analysis, we extracted 336 episodes in which doctors and nurses grappled with finite resources and no tension with safety was apparent. Two authors independently coded 200 of these data episodes according to the level of tension between inclusion and resources (low, high), basis of claim (immediate or recurrent needs, future welfare needs), moral emotions (low intensity, high intensity), and resource allocation (rationing, enabling). Inter-rater agreement was high, and disagreements were resolved through discussion and clarification of the coding scheme. One author coded the remaining fieldnote episodes. When this was completed, custodianship took the form of resource-rationing work in 316 episodes (206 claims for immediate needs, 110 claims for recurrent needs) and resource-enabling work in 20 episodes.

To verify whether these same processes were evident in the doctors’ and nurses’ accounts of their own lived experience, we revisited the interview data. We extracted text segments in which interviewees provided examples of specific instances of claims made by a particular patient and more general descriptions of ED responses to common types of claims, producing 159 interview episodes. Coding classified custodianship as resource-rationing work in 108 episodes (63 claims for immediate needs; 45 claims for recurrent needs) and as resource-enabling work in 51 interview episodes. While this coding focused on the level of the claim, we also coded one instance in the fieldnotes of the ED declaring ambulance bypass. In our view, this was an episode of custodianship in response to extreme inclusion–resource tension at the level of the local place, which was supported by interview data. We present a summary of the coding frequencies of episodes involving the inclusion–resource tension in Table 1. Tables 2 and 3, which appear in the “Findings” section, offer representative data.

**Table 1.** Coding Frequencies for Fieldnote and Interview Data Episodes

Tension between Social Inclusion and Finite Resources		
Low tension	Claims for immediate needs	Fieldnotes = 206 Interviews = 63
	Claims for recurrent needs	Fieldnotes = 110 Interviews = 45
High tension	Claims for future welfare needs	Fieldnotes = 20 Interviews = 51
	Bypass declaration	Fieldnotes / Interviews* = 1
Tension between Social Inclusion and Safety		
Low tension	Claims with known risks (violence, familiar infectious diseases)	Fieldnotes = 69 Interviews = 54
High tension	Claims with unknown risks (Ebola)	Fieldnotes / Interviews^†^ = 4

* There was one instance of bypass recorded in the observational fieldnotes. Multiple interviewees made general comments about bypass.

† Episodes involving the same four persons who made claims on the ED with symptoms suspected of being associated with Ebola were covered in both the fieldnotes and interviews. None of these claims was confirmed as Ebola.

**Table 2.** Representative Data for Microprocesses of Custodianship when Inclusion–Resource Tension is Low

Interaction between Place Claimer and Custodians	Claim Orientation	Emotion	Claim Response
It is early evening. Person (P) presents with back pain and is worried because their parent experienced similar pain before suffering a heart attack. P is assigned to a bed. A nurse and a doctor (Dr R) conduct observations and an initial assessment, including an electrocardiograph (ECG) of the heart. Dr R discusses P’s symptoms and ECG with a senior doctor (Dr S) who agrees more investigation is needed given P has risk factors for a heart attack and anxiety. As P will need to do stress test on a treadmill, Dr S advises, “If P’s got muscular-skeletal back pain, let’s try to get them pain free—otherwise it will be hard to separate any chest pain from the back pain.” They administer pain relief and admit P to ED short stay area overnight. Cardiac tests are run the following morning and show no heart problem. (Fieldnotes)	Basis for claim: Immediate need ED can resolve with usual resources. *(low tension)* Institutional priority: Inclusion by applying “some practical skills to get them better” (D32) and “doing evidence-based practice.” (N25)	Empathic concern for patient’s anxiety. Dr S says in fieldnotes, “We can give them the answer and reassure them.”*(moral emotion; less intense)*	Ration resources (24-hour bed, investigations, staff expertise) to solve problem within reasonable limits. (D14) *(resource rationing)*
Person P presents with right-sided abdominal pain, has tests and pain relief, spends 24 hours in ED short stay for observation, and is discharged with an outpatient appointment for an MRI. Prior to the MRI, P re-presents to ED in pain. Nurse and doctors assess P and organize a CT scan. Dr S tells P, “I don’t think it is appendicitis. If the scan comes back normal, we shouldn’t do anything else because you’re having an MRI tomorrow anyway.” P’s family member is concerned something will be missed. Dr S replies, “The scan will pick up if surgery is needed straight away. If not, I’d prefer to send P home.” When the CT scan is completed, Dr S and Dr R view the images. Dr S concludes, “There’s something unhappy in there. Could be a cyst.” Dr S rings Gynecology to review P. (Fieldnotes)	Basis for claim: Immediate need for which ED is resourced to “set you on the right road” to solve. (D4) *(low tension)* Institutional priority: Inclusion by applying professional expertise and experience to “put together the puzzle.” (D27)	Concern to do the right thing for patient: “let’s work out how we can sort this out further.” (D29) *(moral emotion, less intense)*	Use appropriate resources (ED bed, staff time, MRI & CT scans, specialist knowledge): “using those investigations rationally so we’re not wasting the resource” (D33); “only do tests if results will change the decision.” (Fieldnotes) *(resource rationing)*
Person (P) presents for sixth time this month. Nurse looks up P’s patient management plan and asks, “How can we help you today?” P complains of chest pain. Nurse assigns P to a bed and takes observations, which are normal. Dr S asks P a few questions and does a short physical exam. Since everything is clinically normal and consistent with P’s usual pattern, Dr S moves P to a chair and gets them a cup of tea and a sandwich before discharging them. (Fieldnotes) Junior doctors do not see frequent attenders because they will spend the next three hours and “then there’s no one to see to four other patients that they might have seen in that time frame” (D20) and “you don’t want a junior doctor taking every test known to man for no particular reason every time they come in.” (N21)	Basis for claim: Frequent attendance; “work out what does this patient really need.” (D12) *(low tension)* Institutional priority: Inclusion by using state resources and professional expertise efficiently for public ED to be society’s “safety net.” (D25)	Empathic concern for individual patient in context of other patients’ needs: “start thinking how are we going to break the cycle, how are we going to rationalize it . . . but I don’t think we are any less empathic.” (D15) *(moral emotions, less intense)*	Minimum safe allocation of resources: “Once we recognize those people, we don’t throw a whole lot of resources at them.” (D24) “We’ve tried to put things in place to assist those patients to more appropriately access resources [for their non-emergency health needs].” (N12) *(resource rationing)*
A person walks out of the ED when refused morphine by Dr S because “it’s not the right treatment, even if you’ve been given it before.” A person receives a script for Valium and leaves. When informed of the self-discharge, Dr E tells nurse, “They’re safe. There’s nothing about their [condition] that makes me want to run after them.” A person who presented under the influence of alcohol wants to leave. After checking they are sober enough to be safe, Dr N shows them the exit and moves on to the next patient. (Fieldnotes)	Basis for claim: “history and behavioral patterns.” (D22) *(low tension)* Institutional priority: Inclusion by complying with state laws protecting individual rights.	Concern for patient welfare: “you would like to help, but they don’t really want social work to get involved.” (D7) *(moral emotions, less intense)*	Uses minimum resources if patient is safe to leave: “We’re not going to hold them here against their will unless there is reason to, i.e., they’re a threat of harming themselves or others.” (N2) *(resource rationing)*

**Table 3.** Representative Data for Microprocesses of Custodianship when Inclusion–Resource Tension is High

Interaction between Place Claimer and Custodians	Claim Orientation	Emotion	Claim Response
A disabled person (P) was brought in by their primary carer with “something pretty nondescript, and it became pretty evident that the issue was that the carer wasn’t coping at home. And so, you know, we could provide kind of almost like emergency respite for P so that the carer could just have a night off to get things back in check, have a moment before going back into it. . . . If it prevents the carer from completely decompensating, then I think that’s worthwhile.” (D6) P is admitted to ED’s short stay unit for 24 hours. Nurses do a risk assessment and contact social work and community services so carer can access more support. (Fieldnotes)	Basis for claim: Disadvantaged family needs extra social support. *(high tension)* Institutional priority: Inclusion through improving support for people with disabilities.	Compassion for human welfare and social justice needs of people with disabilities: “we can’t fix social inequalities, but we can . . . do what we can to help.” (D4) *(moral emotions, more intense)*	Exceptional access to ED resources: “A lot of that is very time consuming in an ED . . . [but] you just do it.” (N12) “There’s a lot of services I can plug them into.” (D25) *(resource enabling)*
Person (P) presents to ED. Dr S notices a pattern of increasing presentations for alcohol abuse. P tells Dr S about missing work days and reprimands by boss. Concerned P was “turning into someone who’s just going to drink themselves to death,” Dr S confronts P: “making them realize that they couldn’t legitimize what they were doing—because they were minimizing it.” Dr S connects P with alcohol support service. Weeks later, Dr S sees P on hospital grounds. “P stopped me and thanked me. P remembered the conversation I had with them, and it motivated them to do something about their problem while they still had the capacity to turn their life around.” Dr S smiles. “That was nice. You never know when you affect the course of someone’s life. Most never come back and tell you.” (Fieldnotes, D13)	Basis for claim: Need for extra support for personal empowerment. *(high tension)* Institutional priority: Inclusion by improving ability and opportunity for person to participate in society.	Compassion for inherent value of human life: “you realize what an absolutely privileged position this is to be allowed in to these people’s personal sort of suffering.” (D36) *(moral emotions, more intense)*	Access to extraordinary resources: “alcohol abuse needs more than what ED can offer. . . . ED goes to a certain point, and then after that, refer them on to the appropriate service to continue on and get more of an in-depth care.” (N24) *(resource enabling)*
“Where [ED] can potentially make a huge difference to someone is victims of domestic violence. . . . You can present statistics to them, and reinforce that they’re not the perpetrator, that they’re the victim, and offer a way out and [bring in] the social worker to reinforce those kind of messages. I can think of people that have gone from thinking, ‘I’m going to go back and it’s my fault’ to ‘Shit, he could actually kill me in the future, and maybe I should get out of that relationship.’ . . . And it’s very real having those kind of conversations because there’s a huge change to someone’s life.” (D2)	Basis for claim: Need for extra support for future welfare. *(high tension)* Institutional priority: Inclusion through social justice: “The last thing we want is to treat them like some sort of second-class citizen.” (D37)	Compassion and desire to protect women’s rights and welfare: “no one deserves this” (D22); “give them opportunities to find the way out of the situation.” (D18) *(moral emotions, more intense)*	“We have a lot of great services available that nurses are empowered to access on the patient’s behalf: social workers, domestic violence social worker, community services.” (N10) *(resource enabling)*

Having completed our coding of how the ED was maintained by custodian work directed at the inclusion–resource tension, we shifted our attention to the inclusion–safety tension. Our initial focus was on claims that posed a threat of harm when there was a low level of tension. We conceptualized these as “known-risk claims.” From our fieldnotes, we extracted 51 episodes of known-risk claims involving violence and 18 episodes involving a familiar infectious disease. From our interviews, we extracted a further 30 episodes of violent claims and 24 episodes of infectious claims. As we assembled this dataset, we became sensitized to custodians’ confidence in risk mitigation as a means of managing fear. We labeled this custodianship work as *harm mitigation*. Coding indicated custodianship through harm mitigation for known risks ultimately ended in resource rationing in 118 episodes and in resource enabling in 5 episodes. Refer to Table 4 in the “Findings” section for examples of our coding.

**Table 4.** Representative Data for Microprocesses of Custodianship when Inclusion–Safety Tension is Low

Interaction between Place Claimer and Custodians	Claim Orientation	Emotion	Claim Response
A very aggressive patient who has been stabbed in the thumb is brought in by police. Threat of harm to custodian from physical violence: “a very, very aggressive guy . . . he was fighting away.” (D40) Doctor chooses to stitch laceration while security guards and police hold patient down rather than expose patient to health risk of sedation under general anesthetic, which will also consume a lot more ED resources. (Fieldnotes)	Basis for claim: Immediate medical need with known risk of physical harm to custodian from patient violence. *(low tension)* Institutional priority: Inclusion by compliance with legal obligations of police to seek professional health care assistance for persons in custody.	Some fear of being hit/hurt, “but I just suck it up, clean his thumb while he’s fighting away, and just [stitch] that thumb together and say, ‘Take him to the watch house again’.” (D40) *(managed fear)*	Mitigate harm through (1) four police officers, (2) six security guards, (3) situational awareness and aggressive behavior management training. Allocate minimum resources (staff, bed, equipment, security guards) to mitigate risk, treat laceration, and ensure patient is medically fit for police custody. *(harm mitigation)*
Person (P) with dementia is brought in from a nursing home after behaving aggressively. Dr S and nurses assess P for sepsis infection. P is moved to Short Stay area for observation, with nurses warned to be cautious. Later, P wakes up and punches nurse, who receives emotional support from colleagues and leaves floor. Doctors try to talk to P, who responds with more aggression. Dr S and a resident, three nurses, and two security guards wheel P to ED’s Resuscitation area for high-level care and organize a dose of sedative. (Fieldnotes)	Basis for claim: Immediate medical need with known risk of physical harm associated with illness. Institutional priority: Inclusion through care for the elderly as a disadvantaged group in society. Dr S says, “The nursing home can’t cope. We have to keep P in the ED tonight and get some specialist advice in the morning.”	Fear initially as nurses ask if patient can be sent back to nursing home, but it is able to be controlled. Dr S says, “It’s not safe to let P wake up. It’s quite sad because P would probably be a nice calm old person without the dementia.” (Fieldnotes) *(managed fear)*	Mitigate harm though (1) aggressive behavior management training and situational awareness, (2) verbal warnings for caution, (3) security guard presence, (4) sedation and seclusion. Allocate resources (staff attention and expertise, physical space, investigation services) to balance patient’s care needs with staff needs for safe workplace. *(harm mitigation)*
Person with HIV/AIDS presents with a fungus infection. A senior ED doctor Dr S assesses the patient, runs investigations aided by a junior doctor and two nurses, and contacts an Infectious Disease specialist who comes to ED to consult. Patient asks Dr S several times to be allowed to go home after being treated rather than being admitted to hospital again. Dr S is sympathetic: “I understand you’ll be more comfortable at home and we’ll try to get you home if we can.” Infectious Disease specialist develops a management plan that allows patient to be safely discharged home. (Fieldnotes)	Basis for claim: Immediate medical need with known risk of transmission of an infectious disease. *(low tension)* Institutional priority: Inclusion through equal access to care for member of socially disadvantaged group and by compliance with public health authority protocols for infection control.	Low anxiety because of well-developed infection control protocols. Empathy outweighs concern of “becoming infected myself. I’m more worried about getting the patient home because that’s what they really want.” (Fieldnotes) *(managed fear)*	Mitigate harm through (1) “Contact Precautions” warning sign, (2) personal protective equipment—glove, gown, mask, (3) disposal containers for contaminated supplies, (4) removal of contaminated bed linen and disinfection of environmental surfaces and equipment. Allocate resources (staff, bed, supplies, equipment, specialist input) to mitigate risk and appropriately treat patient’s infection. *(harm mitigation)*

At this point in our data analysis, we began to conceptualize harm mitigation for known risks as an ordinary microprocess of custodian work that maintains the local place of social inclusion. The Ebola virus made visible an extraordinary form of custodianship when custodians experienced “unknown risk” claims. We assembled a data set by extracting all text related to Ebola in our fieldnotes and interviews and gathering the secondary documents we had collected. We compared within and across these different sources of data to discern how Ebola disrupted the ED. Our coding indicated that Ebola aroused *uncontrollable fear* and triggered contests over whether Ebola should be considered a *normal risk* or a *special risk* for which harm must be avoided. A key mechanism in resolving these contests and bringing fear under control was custodians’ moral emotions. Refer to Table 5 in the “Findings” section for examples of our coding.

**Table 5.** Representative Data for Microprocesses when Inclusion–Safety Tension is High

Mechanism	Representative Data
**Unknown Risk**	
Unfamiliar threat	“You are putting yourself at risk. . . . We don’t even know what that risk is. Like it’s not even like you can say the risk is point one of a percent.” (D12) “Ebola—obviously—is a bit different.” (N15)
Lack of confidence in risk mitigation	“And the very real risk of exposing [a doctor or nurse] to it and then their families potentially, and the fact that truly no system in the world is adequately set up for a major pandemic of something as awful as Ebola.” (D2) Senior doctor S walks down to the triage zone to inspect the designated room for assessing suspected Ebola patients. S searches the room and the antechamber. “There’s supposed to be a protocol and I can’t see it.” Locating the protocol attached to the entry door to the antechamber, S reads the single page and shakes his head in dismay. “This is only about the mask and not the equipment. It doesn’t say anything about how to take off the equipment to avoid getting the patient’s blood and vomit on you, which is how you get infected. And what happens if we get more than one Ebola patient? We’ve only got one isolation room.” S hurries to talk to the nurse manager. (Fieldnotes)
Uncontrolled fear	“When people start bleeding from the eyeballs, everyone gets nervous. . . . You’ve got something that carries a high death rate, you can’t stop it, and it seems like it’s contagious. . . . It creates a huge amount of fear.” (D21) A resident R and two nurses assess patient P who presented with flu-like symptoms. After blood samples are taken, P casually mentions that P’s spouse had recently returned from holidaying in Africa. N gasps. “You needed to report that information to the nurse at the Triage desk.” Swearing, R races to the Triage zone to check the list of countries known to be infected with Ebola and is relieved to learn that P’s spouse had not been in an Ebola-infected area. Sharing the story with a group of doctors and nurses on the next shift, R recalls, “I have never been so scared in my life.” (Fieldnotes)
**Contests over Inclusive Custodianship**	
Normal risk & universal custodianship	“It’s our job to see people that are sick that come through the door. . . . I can understand the fear [of becoming infected with Ebola], but that same doctor would be quite happy to walk up to someone who is off his head on drugs, holding a knife. . . . You expose yourself to much greater risk every single day.” (D14) “[Assessing patients] is what we are here to do. . . . If [Ebola] came through the door, it came through the door.” (N24) Nurse N is frustrated that not all staff see the need for Ebola training. “They don’t seem to get it. We’re an emergency department, and everyone has to be prepared to safely assess these patients if they turn up. We all had to see swine flu patients.” (Fieldnotes)
Special risk & selective custodianship	“I see Ebola in the same line as the retrieval work and stuff like that. Retrieval work is a component of emergency medicine care, but not all of us have to do [it]. There’s special training that’s involved for it, and there’s special levels of understanding that are required to do those sort of special roles. . . . Just to say that we can do things [in the ED] is not necessarily that we *all* should be doing things.” (D8) “But we know very well that this is a very dangerous situation—people have died treating patients that have this condition—and so we’ve got to respond in a special way.” (D5) A group of doctors and nurses walk down the corridor of the Acute zone, handing over patients. The conversation shifts to Ebola. A senior doctor S says, “We should follow what the United States does. We train up a small team of about eight doctors and nurses who volunteer to be involved in treating these kinds of patients.” Some of the doctors and nurses nod and agree that it makes more sense to have a small team. S continues, “Who’s going to be more motivated—the person who wants to be involved for conscience reasons or the person who is forced to be involved? The conscience doctor—yes. The forced doctor—no.” (Fieldnotes)
Reflection on constitutive meaning of place and self-conscious moral emotions	“Look, the bottom line is any patient that’s sick and they need to go to hospital, where are they going to go? They don’t come into the entrance foyer and turn up to the volunteer and say ‘I feel sick. Can you call a doctor down from the ward?’ They come here [to the ED]. . . . So yes, the emergency department by default has to have an Ebola response.” (D24) “It’s caused us to reflect and say, ‘Where is our role? What do we do?’ And also perhaps hopefully identify to the hospital that, in the past, it’s been a default option to say, ‘Well, if there is a problem that’s too hard to fix, just send them to ED. They’ll sort it out.’ And I hope that we have helped with a discussion to say, ‘Well, actually if you’d thought about it in advance, you’d realize that ED is not the place for [people pre-identified by health authorities as potentially infected with Ebola]. They should be in another place’.” (D4) The director of the ED chats briefly with some doctors about Ebola and the dissension among senior ED doctors, some of whom argue they should not be compelled to treat Ebola patients. The position of the hospital executive is that ED staff should not be permitted to opt out of treating patients, whether for Ebola or some other illness. He says, “The hospital executive isn’t the enemy on this one. There is no enemy here. We don’t get to pick and choose which patients we treat.” (Fieldnotes) “They’re entitled to the best care that we can give without any sort of discriminatory barriers.” (N6) “Essentially they’re no different from any other patients, and if they come through our door, we have a responsibility to see them.” (D9)
Restoration of ordinary microprocesses of custodianship work through harm mitigation	“If you’re looking after [an undifferentiated Ebola patient that presents to ED], you take your clothes off, chuck them in a plastic bag, shower, go home in scrubs, and protect yourself. . . . Just being sensible.” (N21) “I’m happy to deal with [an undifferentiated Ebola] patient in the area they’ve designated and put on the gear that we need to put on. Often these patients aren’t too sick. They don’t really need too much doing except vital signs, a bit of Paracetamol and some blood tests. . . . But that quarantine patient who was on with the radar—those patients should not be coming to the ED. Someone who’s a known entity out there should be going straight to the Infectious Diseases facility . . . whereas I’m happy to see anyone who walks in off the street about an issue.” (D7) At the end of the night shift, Doctor D hands over to the morning shift of doctors and nurses and provides an update on yesterday’s suspected Ebola case. “It got completely diverted away from the ED since health authorities knew about it. The person had been in home quarantine.” Smiling, D continues. “That was good. The patient didn’t need our skills so it kept the ED free to focus on other patients who did.” (Fieldnotes)

In the final stage, we developed a process model that theorizes how a place of social inclusion is maintained by custodianship that connects the levels of societal institution and local place in responding to claims and managing value tensions. The robustness of our model was increased by triangulating across multiple data sources, using dialogue and debate in research team meetings to arrive at the most credible interpretations, and debriefing with fieldsite participants to verify interpretations in the context of their experience (Denzin and Lincoln, 2000).

## Findings

Our data analysis shows that the public hospital emergency department (ED) we studied can be considered a place of social inclusion across nested levels of societal institution and local place. At the level of society, the Australian government funds and regulates public hospital EDs to provide universally accessible medical care to all citizens with acute needs. As one doctor put it, “The public hospital ED is the ultimate environment . . . [where] it’s a privilege to provide a service to everybody” (D38).^2^ In contrast to the pay-for-service ED in private hospitals, the public ED is a place of social inclusion where “everyone is equal” (N6) and “everybody deserves the same sort of entitlements and rights as everybody else” (D41). For Australian people in marginalized and vulnerable groups—such as the homeless, mentally ill, drug or alcohol addicted, and socially disadvantaged—the public ED is often “the one place where these guys can get looked after” (D31). Public EDs in Australia “are the safety net for vulnerable people . . . whatever’s going on [in society] we become that place” (D21). At the same time, there is broad recognition that public EDs provide care that is not only socially inclusive but of high quality. A hospital executive at our fieldsite ED said, “If I was really sick I would like to be in here, which is always the test!” (M4).

Our findings suggest that the institution of a place of social inclusion is actualized at the local level in geographically specific EDs located in towns and cities across Australia. The societal value of universally accessible health care for all Australians inheres in our fieldsite ED as a local place where any citizen with acute health needs can present to make claims. As a local place of social inclusion, the ED is expected to be “the saving place for so many people who come [here to make claims] for all sorts of reasons” (D1). Yet the data indicate that the combination of societal institution and local place has consequences that make this accomplishment difficult. The ED is geographically and materially bounded because the government has assembled a finite stock of resources—staffing, beds, equipment, diagnostic technologies, and other supplies—at the local place to respond to citizens’ claims. This creates a tension between the institutional value of inclusion and local resources. A nurse described this value tension: “Everyone is entitled to health care, but you’ve only got this much resource and you’ve got this [gestures with hands to indicate volume of people presenting to the local ED]—how do you match them?” (N6). A second value tension concerns local safety. At the societal level, the ED as a place of social inclusion “should at least be a safe place to come” (fieldnotes). But our data show that translating this value at the local level into “an open door policy to members of the public” (fieldnotes) exposes the fieldsite ED to risks of harm arising from infectious diseases and acts of violence.

These value tensions render EDs precarious as places of social inclusion because the societal value of universally accessible health care can only be realized by consistent actions of inclusion at the geographically specific ED. Our analysis shows doctors and nurses tried to protect this institutional value through ongoing local-level custodian work. Because doctors and nurses believed “the basic ethos of trying to provide equal health care for all is something we should fight for” (D45), they invested effort in custodian work to resolve value tensions and maintain the ED as a place of social inclusion on top of their normal work as organizational employees and members of the medical and nursing professions. Our findings indicate that custodianship directed at value tensions is “extra” work performed over and above the professional work that characterizes an ED more narrowly as a place of medicine.

### Local Resources and Maintaining the Place of Social Inclusion

Persons who presented to our fieldsite ED were exercising their societal-level right to make a claim for access to the staff, beds, and other material resources at this local place of social inclusion. To insure social inclusion, custodians should “never say no . . . never refuse treatment” (D33). Yet responding to all claims at the highest level of service would quickly exhaust finite resources, leaving claims unmet and rendering the ED unable to fulfill its social purpose. We found that doctors and nurses engaged in two processes of custodianship to manage this inclusion–resource tension: resource-rationing work and resource-enabling work.

#### Resource rationing

Doctors and nurses most commonly responded to claims by rationing the ED’s finite resources. As custodians, they were “constantly thinking about whether the patient needs these resources” (fieldnotes) and mulling over “here are our competing demands—how do we organize our resources?” (D46). They sought to assess claims efficiently and “activate the resources that are needed” (D13), being “very judicious in that use” (D4), as this example shows:

Person P presents to ED. Triage nurse asks, “What brings you here today?” P describes vomiting and abdominal pain. P is assigned a bed. Nurse N inserts a drip and takes blood samples. Senior doctor, Dr S, notices that P “looks pretty sick,” examines P, and prescribes anti-nausea medication. N offers comforting words, and Dr S orders a CT scan to check for an obstruction. A radiologist suggests using a contrast dye with the CT scan, but this means P will need a bed for longer, and Dr S is not convinced the dye is medically warranted. After the scan, Dr S moves P to the ED’s short stay unit for 24 hours with a plan that if the vomiting and pain settle down, P will be discharged and given a follow-up appointment with a hospital outpatient clinic. If symptoms persist, P will be reviewed by surgeons for an operation. (Fieldnotes)

This example illustrates how custodian work occurs through resource rationing. The doctor and nurse evaluate the basis for the claim as an immediate health need. Perceiving that the ED has adequate resources to resolve this need, they experience low tension between the institutional value of social inclusion and the local ED’s finite resources. They allocate appropriate, but not excessive, resources and emotional energy to meet the need underlying the claim (staff time and expertise, investigation, equipment, 24-hour bed, empathy), which maximizes the ED’s ability to respond to other claims. Had this been a pay-for-service private ED rather than a place of social inclusion, the doctor would not have had to think about how “ordering a CT scan may delay another CT scan for another patient—you have a different [responsibility]” (D44). A doctor who worked in both the fieldsite ED and a private ED explained, “I will make different decisions on what I do in the public sector and private sector because I know there is different access and availability in the . . . resources” (D34).

Table 2 presents examples of these microprocesses in which custodians apprehend a claim as an immediate or recurrent health need, experience low tension between social inclusion and finite resources, and meet the need by rationing resources. Examples of claims for immediate needs included new symptoms that a person was experiencing such as back pain, acute exacerbations of pre-existing conditions such as diabetes, and diagnostic puzzles such as multiple sclerosis. Claims for recurrent needs involved the repetition of a past illness or behavior, such as unchanged chronic illness or frequent attendance for non-emergency claims. Doctors and nurses responded to claims for recurrent needs by allocating “the bare minimum” (D20) resources because they were “obliged to sort out [the claim] enough to allow the patient to go home in some sort of safe and dignified manner” (D22). When a patient’s needs can be adequately met with the usual attention to rationing resources, custodian work involves moral emotions of low intensity because custodians “feel comfortable about how they can deliver care within those constraints” (M2).

Our data suggest that resource rationing balances the inclusion–resource tension in a way that upholds the institutional value of social inclusion at the local place by allowing custodians “to try and treat as many people as possible as well as possible” (D27). According to our informants who have comparable experiences of working in pay-for-service EDs in private hospitals and in other departments in public hospitals, this process of resource rationing is distinctive to their custodian role at the fieldsite ED. Participants reflected that resource-rationing work is “a different reality” (D33) for medical and nursing professionals employed in a public ED because no other place of medicine in Australia has responsibility for accomplishing the value of universal accessibility to medical care. That is, Australian citizens “know that the emergency department never shuts and know that the ED is never going to turn us away” (D21). EDs in private hospitals, which are “essentially business” (D29), do not have this responsibility. Nor do other departments in public hospitals because they are not “the initial point of contact for people coming from outside” (R8). When working in those departments, participants said, “You’re not thinking about the limited resource” (D32) in the same way as in the ED. With “totally different flow, totally different drivers, totally different demands” (D34) in a public ED than in other places of medicine, resource-rationing work to balance the inclusion–resource tension is distinctive to a public ED as a local place of social inclusion.

#### Resource enabling

Our analysis revealed that not all claims on the ED’s finite resources could be addressed adequately by custodians performing resource-rationing work. Sometimes custodians made judgments that a claimant’s needs extended beyond an immediate or recurrent health problem to future-oriented needs, as vulnerable people presented to the ED seeking support to change their life circumstances. When responding to these claims as custodians, doctors and nurses experienced a high level of tension between the institutional value of social inclusion and the ED’s finite resources as a local place: “The ED is an opportunistic place for some of this stuff to happen . . . around social disadvantage, but it takes a lot of health resource to do that” (D20). A doctor described the tension over resource allocation when responding to a future-oriented claim: “All of us have got this feeling of social justice, but that’s the problem with this sort of stuff—everything else just ground to a halt because I couldn’t do everything for this one patient and all the rest in the ED, but that’s what it takes” (D8). Responses to claims that custodians judged as being made by persons who were especially “vulnerable went above and beyond normal [allocations]” (R10) from the ED’s finite supply, creating heightened value tension:

Elderly person (P) presents with back pain. Questioning by a senior doctor (Dr S) reveals P has had multiple car accidents and has advancing dementia. A team of six people—Dr S, a junior doctor, social workers, and community services—spend the entire day organizing a hospital admission, home support, and removal of driver’s license. For the two ED doctors, the intervention to keep “the most extraordinary complicated social circumstance from advancing to complete disaster” dominates everything else going on in the ED, where other doctors and nurses work as best they can to cover for their absence. Dr S, who stays to resolve some issues for an extra two hours after the end of shift, describes how P’s family “went home just sobbing because someone had made an effort to try and sort it out.” Emphasizing “the emergency department was a great site for it to happen,” Dr S adds, “But it took a huge amount of work. That’s a lot of health resource.” Dr S tells the junior doctor, “That’s probably the greatest intervention you’ll achieve as a junior in an emergency department. Much more so than fixing a broken arm. . . . This is far more important to have achieved.” (Fieldnotes, D20)

This example illustrates the microprocesses through which custodians reconcile a high level of tension between the institutional value of social inclusion and local resources. The senior doctor judged the basis for the person’s claim as a need involving social justice and human welfare. This need aroused intense moral emotions, with the doctor deeply concerned for the patient and family and motivated to activate extraordinary resources to accomplish the highest values of the ED as a place of social inclusion. Rather than the ED’s finite supply of resources constraining the claim response, the doctor instead enacted his role as custodian by interpreting local resources (bed, staff time, social workers’ knowledge, community services) as enablers of a more enduring intervention for the family’s welfare.

Table 3 presents other examples of custodians evaluating claims as human needs that exceed ordinary resource allocations, experiencing high tension between inclusion and resources, feeling intense moral emotions, and activating resources as enablers. Examples include claims by victims of domestic violence and by other vulnerable people judged to be in crisis and in need of “community services to help support them” (N12). Claims can also trigger resource-enabling responses when custodians judge a person with an addiction as sincerely wanting help to “change in their trajectory” (D3). Finally, claims for end-of-life care prompt resource enabling when the ED provides extraordinary resources to honor human dignity at the end of life. For custodians, the human needs underpinning these types of claims arouse intense moral emotions of empathic concern: “If you’re not upset by grief . . . or a tragic story, you need to go and get another job” (N1). Some claims aroused moral emotions of such intensity that “there are ones that will live with me forever” (D8).

Our data suggest that these processes of resource-enabling work are distinctive to custodianship of public EDs. Reflecting on their experiences being employed in both public and private EDs, several research participants explained that there is no “naïve separation” (D16) between acute medicine and social disadvantage in the fieldsite ED, as opposed to in a private ED. Some participants asserted that other specialist departments in the public hospital could also separate social disadvantage from medicine to some extent because the ED provided a buffer as “the first port of call” (R3, R4) between community and hospital. Resource-enabling work allowed custodians to uphold the highest ideals of a public ED as a place of social inclusion for the most needy citizens: “rightly, the community should expect more from us” (D37). While it was easier for custodians to allocate more resources to these types of claims when the ED was not overloaded, custodians tried to keep focused on “making the right decision [about the person’s resource needs] each time . . . even if the place is heaving” (D39). As our field observations demonstrate, if resource-enabling work is “the best we can do [for a person’s needs], we run around like crazy trying to achieve that” (N23).

#### Place disruption

The data show that custodianship through resource-rationing work and resource-enabling work maintained the fieldsite ED as a local place of social inclusion in two important ways. First, these forms of custodianship resolved the inclusion–resource tension at the level of individual claims so that local custodians could respond appropriately to a person’s health needs. Second, they managed the inclusion–resource tension at the local place by ensuring that the ED remained open. Because “the reality is we don’t really have a lot of control about who comes through or how many people come through the front doors” (R7), there is potential for the volume of claims to completely overwhelm the ED resources available at the local place. If this reaches “a crisis point,” state regulation and associated processes permit a local public hospital to “deem that their ED is full and unsafe, and they will redirect ambulances to other hospitals . . . [to allow] time to just decant patients” (N3). While ambulance bypass safeguards the institution of the public ED as a place of social inclusion at the societal level, it means the ED as a local place is temporarily closed to citizens’ claims.

Our observations contained only one example of the fieldsite ED declaring bypass, along with numerous examples of busy shifts when doctors and nurses said “it was a badge of honor” that they had worked hard to avoid bypass (fieldnotes). Hospital managers also told us that custodians in the ED “don’t want to go on bypass, they want to manage it” (M4). According to our data, doctors and nurses at our ED perceived ambulance bypass as a disruption of the local place of social inclusion. In their eyes, by closing off the local place from its “value relationship with the community” (D46), bypass disrupted the meaning of the ED as a universally accessible place for people with acute health needs. To protect this value, doctors and nurses took pride as custodians in using resource-rationing work and resource-enabling work to avoid bypass whenever possible to keep the fieldsite ED open:

Bypass—that’s the thing we work hard not to do. I guess sometimes when it does happen . . . you do feel a little angry [and disappointed that] . . . clearly you aren’t able to provide the service that an emergency department is supposed to be able to provide to its patient catchment [population]. . . . Bypass isn’t something that we do very often. You just keep beavering away. (D45)

Our hospital very rarely does that. Almost never. Our doors are always open. It’s like a pride thing. We can handle anything. (N3)

### Safety and Maintaining the Place of Social Inclusion

In their role as custodians of the ED as a place of social inclusion, doctors and nurses confronted a second source of precariousness. This arose from tension between the societal value of social inclusion achieved through universal accessibility for all citizens and the safety of the local place. At the societal level the public ED is intended to be “a place of safety,” but at the local level being open to everyone in the community means the “ED is always a great entry point . . . for risk” (D12). Our participants pointed out that compared with private fee-for-service EDs, public EDs had responsibilities for a wider “spectrum of humanity . . . [so] there’s a significant risk” (D2). At our fieldsite, tension between inclusion and local safety arose when a person making a claim posed a threat of harm to custodians and citizens. Our analyses found that doctors and nurses engaged in two processes of custodian work to manage this inclusion–safety tension: harm mitigation and harm avoidance.

#### Harm mitigation

Our data show that certain people who made claims for access to health care at our fieldsite ED posed known risks of harm to staff and other patients. Doctors and nurses classified the threat of harm as a known risk based on their familiarity with the threat and their confidence that the risk of harm could be mitigated.

The most common known risk was violence. Persons making claims could act violently due to physiological (e.g., dementia, brain injury), psychological (e.g., mental illness), toxicological (e.g., alcohol abuse, drug overdose), or behavioral causes. Doctors and nurses evaluated violent patients as a familiar threat because “we deal with it on such a regular basis” (N2) and “have a system that we’re confident to deal with them” (D16). Risk mitigation mechanisms included security guards, verbal de-escalation, security cameras, and patient isolation and sedation. Because violence was a known risk, doctors and nurses reported being able to manage their fear of harm to the extent that they could respond inclusively to claims. Our fieldnotes show, for example, nurses being shaken up after being verbally abused by a patient but continuing to administer care, and doctors examining angry drunk people even though “they look ready to take a swing at me” [fieldnotes].

Another threat of harm that custodians classified as a known risk was associated with infection. Persons with blood-borne viruses (e.g., hepatitis C, human immunodeficiency virus/HIV) and diseases transmitted via droplets (e.g., influenza, whooping cough) and/or airborne routes (e.g., measles, tuberculosis) posed the highest risk of infection for doctors and nurses. Custodians classified infectious diseases that had a well-understood and familiar disease process as known risks. For example, doctors and nurses “understand the flu a bit more and have had experience with it” (D16), occasionally treat cases of measles and tuberculosis (fieldnotes), and “could get a needle stick every day from a HIV patient or a hep-C patient . . . we’ve got used to those risks” (D11). Familiar infectious diseases had evidence-based risk mitigation procedures that doctors and nurses trusted, such as infection control precautions, personal protective equipment, warning signs, quarantines, and staff immunization. Because they “knew how to deal with infectious diseases that came to the front door” (D4), custodians could resolve the inclusion–safety tension when responding to claims:

If these patients come here seeking help, you see them and you assess them properly [while mitigating the known risk of harm], and then you make sure that they’re okay and you activate the resources needed for them. . . . I’m nervous about it when you walk in to see someone who’s angry and snarling and spitting, where you’ve got a much greater risk of actually getting a transmissible disease. But you do what you have to do. . . . Are you going to refuse to see them because your chance of getting harmed is much higher? Of course not. (D13)

As the example above illustrates, when doctors and nurses classify a person making a claim as a known risk, they perceive tension between the safety of the local place and social inclusion. Despite feeling “slightly excited and scared about what’s coming up” (N2), they are able to manage their fear sufficiently to perform their role as custodians by mitigating risk and then activating resources to meet the human need underlying the claim. The above example also illuminates how managing fear is bolstered by custodians’ moral emotions related to a sense of concern for doing the “right thing” to meet the needs of the patient and society more broadly. A nurse, who had recently been cut with a knife when responding to a claim by a suicidal patient, explained: “My Achilles heel in those situations is trying to help the patient, so I’ll put myself in a bit more danger” (N24).

In one compelling example from our fieldnotes, we observed an elderly dementia patient punch a nurse in the face, sparking fear among the ED team responding to the claim and triggering a self-protective instinct to withdraw access. Nurses implored, “Can’t we just send him back to the nursing home tonight?” Our fieldnotes show that moral emotions associated with not wanting to violate their custodian responsibilities to the community (“the nursing home can’t cope”) and empathy for the patient (“it’s quite sad”) helped subdue fear. The treating team resolved the inclusion–safety tension by mitigating the known risk through sedation, which allowed them to safely keep the patient in the ED overnight until specialist services could provide support the next day. Table 4 presents other examples in which custodians classify a claim as a known risk, experience low inclusion–safety tension and manageable fear, and are motivated to take action by mitigating the harm and then rationing or enabling resources to meet the person’s need. Harm mitigation maintains the ED as a place of social inclusion by keeping the local place safely and universally accessible to citizens.

#### Harm avoidance

Custodianship through harm mitigation for claims that posed known risks, combined with resource-rationing work and resource-enabling work, usually maintained the fieldsite ED as a place of social inclusion at the local level. However, our data related to Ebola, which are illustrated in Table 5, reveal that custodianship can break down when claims carry unknown risks of harm, amplifying tension between the value of social inclusion and safety of the local place. After the World Health Organization (WHO) in 2014 warned of the threat of a pandemic through international travel of persons who had lived in or visited Ebola-infected regions, the public hospital where we collected our data was designated by the government as one of Australia’s “Ebola response and treatment hospitals.” Thus our fieldsite ED became the local place that public health authorities, who monitored arrivals from West Africa and imposed home quarantines, would send suspected cases of Ebola for assessment. The ED was also the designated local place where other unwell persons who may have come in contact with Ebola through travel or contact with travelers could present for assessment.

Government authorization of claims on the local place by persons potentially suffering from Ebola carried an unknown risk of harm for doctors and nurses as custodians of our fieldsite ED. The threat was unfamiliar because Ebola was “a new exotic disease” (N24) and there was insufficient “experience of it in a Western context to even know what the risk is” (D12). Infection controls were unproven. Pointing to incidents of health care workers becoming infected, doctors and nurses saw Ebola as “extremely contagious” (N15) and “an illness that targets us so there is that perceived threat” (D17). This undercut confidence that risk could be mitigated: “I don’t know that we’re confident in our systems with Ebola” (D16).

The unknown risk of potential Ebola claims aroused far more intense fear than doctors and nurses customarily experienced in their everyday work: “There’s obviously things unknown about it [Ebola], so there is a fear factor and rightly so” (D17). In our fieldnotes and interviews, custodians described their emotions in anticipation of persons making claims for health needs associated with Ebola as “fear,”“angst,”“being scared,”“raw emotions,”“terrifying,” and “anxiety.” Nurses noted “they call it the carer’s disease” and said “it’s very scary for me as a nurse” to be at risk from an infectious patient (N16). The fieldnotes capture how doctors and nurses struggled to control the fear aroused by Ebola as an unknown risk:

World Health authorities have just announced that a second nurse has become infected while caring for an Ebola patient in a U.S. hospital. In the ED’s central work area, doctors and nurses are alarmed that hospitals in the first-world could not prevent Ebola transmission to health care workers wearing Personal Protective Equipment (PPE) and following protocols recommended by the Centers for Disease Control. “So how did they get sick if they were wearing the correct PPE?”“Do we really understand how Ebola is transmitted?” Throughout the shift, doctors anxiously check for updates and review the ED’s stocks of protective clothing, concerned about its effectiveness. In a follow-up interview, a doctor justified their fears: “Suddenly two nurses actually catch it in America, and then the staff suddenly can see we’ve got a right to be scared.” (Fieldnotes)

Fearful doctors and nurses sought information to assess the “real risk” of harm from Ebola claims. Information was accessible through mass media coverage and social media networks among emergency physicians (fieldnotes). WHO and the U.S. Centers for Disease Control and Prevention disseminated information, and government policymakers in Australia and hospital executives sent out communications and updates. These state-endorsed sources offered scientific data and projections on Ebola and reported on infection control and border protection protocols. Conversations about Ebola “bubbled” (D8) among doctors and nurses: “You couldn’t go anywhere without hearing the word ‘Ebola’ when you’re in the tea room or the corridor. It was just an Ebola fest” (D17).

Over time, evaluations of the harm posed by Ebola fragmented into two risk categories among doctors and nurses as custodians of the local place. The first category evaluated Ebola claims as a *special risk*. While acknowledging that Australia’s border protection protocols would likely prevent a person in the highly infectious “wet stage” of the virus from reaching the local place, doctors and nurses in this category noticed cues from multiple information streams that two nurses in the U.S. had become infected and bracketed these cues as important. A doctor explained, “If those two nurses hadn’t caught it, I think [the perceived risk of Ebola] would be totally different” (D16). These cues focused sensemaking attention on differences between Ebola and more familiar threats. Ebola’s high transmission risk, incurability, and high mortality rate were interpreted as a different sort of risk from that posed by violence and other infectious diseases. Ascribing significance to these differences produced the evaluation that claims by persons with Ebola represented a “special” risk.

The second risk category evaluated Ebola claims as a *normal risk*. These ED staff noticed cues from state-endorsed sources that controlled “the hysteria of ‘Ebola is coming to kill us all’” (D14). These cues drew attention to contextual differences between Australia, a first-world nation with protected borders and advanced health systems and infection controls, and West Africa, a third-world region with tribal practices and environmental conditions that accelerated Ebola transmission and mortality. A nurse explained, “The government [here] has put out precautions and assessed it and worked it out, and obviously you’ve just got to trust in them that that is going to contain it” (N24). By contextualizing Ebola, these custodians became “more calm” (fieldnotes) and less “caught up in the emotion of it all” (D18). Sensemaking concentrated on similarities between Ebola and known risks (fieldnotes), and “it became pretty obvious that this [situation] was nothing other than normal” (D24) and nothing special to be “worried about” (N15). Claims for Ebola were categorized as a normal risk, equivalent to violence and other infectious diseases already accepted in custodian work:

The chance of you being killed or assaulted [by a violent patient] is much higher than if you walk into a controlled environment with full protective gear on. Nothing is going to be transmitted. (D14)

Fragmented risk categories created contests among custodians about inclusive custodianship for potential Ebola claims. Categorizing Ebola as a normal risk justified maintaining ordinary custodianship through harm mitigation. A doctor argued, “Why do we make a special thing for Ebola patients? I don’t understand it” (D3). If the “real” risk of infection was negligible, all staff could be trained to a level of proficiency to engage safely with Ebola claims: “This should be no different for us [than] treating someone with a febrile neutropenia [fever with signs of infection]” (D7). In contrast, categorizing Ebola as a special risk problematized custodianship and justified custodians avoiding harm by not responding to a claim. If Ebola is an exceptional risk, then “we’ve got to respond in a special way” (D5) and respect that “some people have a view they don’t want to be involved at all” (D1). Wanting to opt out of responding to Ebola claims, some custodians proposed covering extra shifts in the ED while a small group of doctors and nurses self-selected to be “intensively trained and regularly practicing as Ebola rapid responders” (fieldnotes).

#### Place disruption

Our data show the contest between the normal risk (harm mitigation) and special risk (harm avoidance) approaches to custodianship for potential Ebola claims disrupted the taken-for-grantedness of the fieldsite ED as a local place of social inclusion. With some doctors and nurses advocating withdrawing their normal custodian responsibilities for universal access, custodians of the local place were no longer fully embodying the values that defined the institution of a public ED at the societal level: “In ED, we don’t get to pick and choose our patients” (N6). For custodians, the taken-for-grantedness of the fieldsite ED as a universally accessible place for people with acute health needs, as well as their own identity as the local protectors of that institutional value, was disrupted. Articulating this disruption, a doctor said, “There’s that real challenge to the way we’ve always seen ourselves in ED as ‘We’ll see anyone, we’ll help you, we’ll heal you’” (D11).

Our data show that during the disruption of the local place, some doctors and nurses felt embarrassed that custodians were not living up to the role expectations that society had entrusted to them. A doctor explained, “Hysterical responses to Ebola were a little embarrassing . . . [because] my identity as an emergency physician in a public ED is that if someone needs care then you give them care—you don’t pick and choose who you see” (D13). Others noted that it was “disappointing” (D24) and “embarrassing . . . for people to say we’re not here for this. Aren’t we? I’m pretty sure we are” (D14). Individuals contemplating the violation of their custodian responsibilities by not responding universally to Ebola claims wrestled with both fear and self-conscious moral emotions like shame:

I don’t even know if I’d be walking in there [to treat a person who might have Ebola], but by the same token that’s not the way we’re built in the public ED. We can’t say, “Come here if you’re sick and we’ll see you and we’ll sort you out unless you’ve got Ebola.” . . . And I think that’s what’s caused that real level of angst, and it’s challenged the way you perceive yourself [as a custodian] much more so than anything else. (D11)

These emotional contests over custodianship of Ebola claims came to a head at a meeting of senior ED doctors, a member of the hospital executive team, and the Infectious Diseases Department. The ED director organized the meeting to “let people vent their spleen” in a facilitated forum about how the local place should respond to Ebola claims (fieldnotes). ED doctors used the meeting as a “useful tool . . . for having those fierce conversations” (D20) about their differing views of Ebola risk and custodianship. The meeting was emotionally charged. Doctors expressed “forcefulness of opinions . . . that showed how diverse views are” (D18).

Our analysis reveals that the robust debates in this meeting, which were continued in follow-up meetings among emergency staff, aided recovery from the disruption by clarifying the constitutive meaning of the public ED as a place of social inclusion. These debates “really crystallized” (D4) for custodians two constitutive elements that connected the societal institution of the public ED with the local place. First, the ED as a place of social inclusion was constituted by the salient attributes of persons making claims. If a person was acutely sick and undiagnosed with an illness, “then absolutely the ED’s the right place for them” (N20). Second, the ED’s societal-level meaning as a place of social inclusion was constituted by local responses to claims that upheld “the general principles of people having equal access” (D1). Custodians agreed “the role of a public ED . . . is to treat everyone exactly the same” (N14). Thus, when someone who may be symptomatic with Ebola “walks in off the street” (D7), custodians of the fieldsite ED as a local place must uphold universal access.

Recovery of the constitutive meaning of the place of social inclusion motivated custodians “to come up with a local emergency department–based solution to this potential risk” (D5) and protect the ED as a local place through harm mitigation. Custodians devised procedures to mitigate risk in the Ebola isolation room, sought advice from infectious disease experts on protective equipment, and encouraged training but respected individual choice: “We’ll let those who want to participate, participate” (D22). Most senior doctors and nurses undertook training and grew more confident that the risks of Ebola could be mitigated. A few custodians elected not to train. Although Australia had no confirmed cases, the ED received a small number of claims by persons suspected of being infected with Ebola.

Custodian responses to these claims confirmed that the ED was restored as a place of social inclusion following the disruption. Claims were made by persons who turned up at the ED with a fever and who may have had exposure to Ebola through international travel but whose symptoms could stem from other illnesses such as malaria. Mitigating the risk of harm and managing their fear, doctors and nurses assessed the needs underlying each claim and activated ED resources—staff, isolation room, blood tests, investigations—to respond safely and appropriately. A nurse who cared for a few suspected Ebola patients said, “If someone came in with symptoms . . . we made sure we did everything right” (N21). Through this harm-mitigation work, custodians preserved the societal values of the ED as a universally accessible place of social inclusion at the local level:

It’s our role to see everything. I guess there was a lot of concern initially . . . because in [this city], we are the Ebola hospital . . . but our concerns and our emotions were contained [over time]. You can’t ban the community just because you have certain concerns. . . . Public emergency departments are a place if you’re sick you can come to Emergency and we’ll look after you. . . . It’s an important role. (D25)

### Process Model of Maintaining Places of Social Inclusion

**Figure 1.**
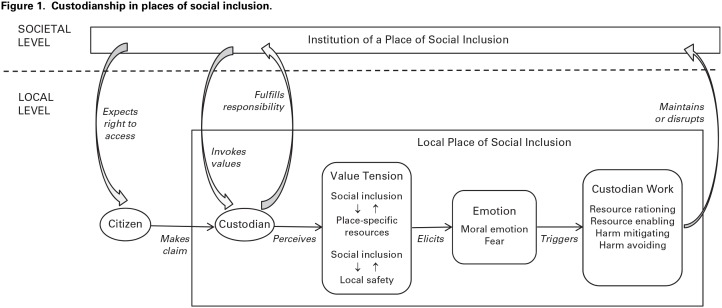
Custodianship in places of social inclusion.

The theoretical model we developed from our findings is presented in Figure 1. The combination of place and institution creates a nesting of geographically specific places of social inclusion at the local level within the institutional level where these places are regulated, valued, and given meaning in society more broadly. The democratic state establishes and supports places of social inclusion as institutions to meet citizens’ needs for universal access to essential services. Our model shows how the institution of a place of social inclusion can be maintained through microprocesses of custodianship at the local level. Every time a citizen exercises their right to universal access and presents at a local place of social inclusion to make a claim for services, custodians have a responsibility to respond. Custodians are also deeply committed to the institution’s values of social inclusion. When a custodian perceives tensions between the institutional value of social inclusion and the local place’s ability to actualize the value, these tensions elicit emotions and motivate multiple forms of custodian work that maintain the local place of social inclusion.

As illustrated in Figure 1, custodians of a local place of social inclusion experience tension between accomplishing the societal value of social inclusion through universal access for citizens and working with the finite resources available at the local place for responding to claims. Perception of an inclusion–resource tension evokes moral emotions such as empathy of varying intensity, motivating custodians to reconcile the tension by engaging in resource-rationing work and resource-enabling work. If custodians are unsuccessful, the inclusion–resource tension can become so unbalanced that universal access breaks down. By allocating resources in ways that are or are not inclusive of all claims, custodian work at the local place maintains or disrupts the institution of a place of social inclusion at the societal level.

Custodians of a place of social inclusion at the local level can also perceive tension between the institutional value of universal access and the safety of the local place. When custodians evaluate a citizen’s claim as carrying a known risk of harm, the inclusion–safety tension arouses moral emotions and manageable fear that motivates custodians to engage in harm-mitigating work. By safely protecting universal access at the local level, this work maintains the place of social inclusion as an institution. In contrast, when custodians evaluate a claim as carrying an unknown risk, the inclusion–safety tension arouses fear that is difficult for custodians to control and motivates a desire to keep the local place safe through harm-avoiding work. Because failing to respond to a claim deviates from a custodian’s responsibilities and value commitments to upholding universal access, moral emotions are also elicited. If moral emotions reduce fear to a manageable level, custodians find ways to mitigate harm and protect universal access at the local place, thereby maintaining the institution of a place of social inclusion at the societal level. But if custodians’ fear outweighs moral emotions, they feel justified in engaging in harm-avoiding work to protect the safety of the local place. In this case, access is denied for some or all citizens who want to make a claim at the local place, disrupting the place of social inclusion as an institution.

## Discussion

We bring together the literatures in humanistic geography, sociology, and institutional theory in organization studies and make a significant contribution by proposing a place of social inclusion as a special type of institution. Our concept of a place of social inclusion is anchored in scholarship about public places of democracy that are accessible to all citizens (Altman and Zube, 1989; Carr et al., 1992), local places that are geographically bounded, material, and meaningful (Tuan, 1977; Gieryn, 2000), and societal institutions as nested multi-level systems (Scott, 2014). Although the possibility that places of social inclusion exist can be inferred from these previous writings, their distinctive characteristics have not been conceptualized and integrated into theoretical understandings of institutions until now. The literature pointed us to the initial definition of a place of social inclusion that we sketched at the beginning of this paper. Our empirical study of a public hospital emergency department now allows us to deepen and elaborate our understanding of the defining societal-level and local-level characteristics of a place of social inclusion as an institution. We contend that places of social inclusion are distinguished as institutions by the following characteristics: (1) establishment at the level of society to accomplish values of social inclusion by providing citizens with universal access to services for essential human needs; and (2) endowment of geographic sites at the local level with material resources, meaning, and values as places where citizens in need can make claims.

A consequence of these two defining characteristics is the associated value tensions between universal accessibility and the finite resources and safety of the local place. We suggest that our theoretical model of how these tensions create conditions for custodianship may be generalizable beyond public emergency departments to other places of social inclusion. To illustrate, we offer an example of the model’s application to public schools, which are “pervasive institutions” in society and local communities (Longhofer, Negro, and Roberts, 2019: 223). While the “egalitarian ethos” of education-for-all exists at the societal level (Domina, Penner, and Penner, 2017: 311), students live in neighborhoods with particular demographic, economic, and racial characteristics and make claims for access to education at their local public school (Arum, 2000; Longhofer, Negro, and Roberts, 2019). Principals and teachers in local public schools at times perceive tension between the societal-level value of universal access and the resources available to meet student needs at their particular school, such as classrooms, staff, technology, and equipment (Arum, 2000). Our model suggests this value tension motivates some of them to act as local custodians by rationing resources to meet students’ ordinary needs and enabling resources for vulnerable and/or gifted students. We do not assert that these custodian judgments will always or necessarily accomplish the highest values of social inclusion in terms of social justice and human empowerment. On the contrary, our model contends that whether a student’s claim for education at a neighborhood public school elicits resource-rationing work or resource-enabling work depends on the subjective judgments and moral emotions of local custodians.

Continuing our model’s application to public schools, custodians may also experience value tension between societal-level expectations of universal access to education and “school safety and order” at the local place (Cornell and Mayer, 2010: 7). Safety threats can arise from the spread of infectious diseases like measles (Rowan, 1982) when claims for access are made by unvaccinated students posing risks of harm at local schools with insufficient herd immunity (Becker et al., 2016). Our model explains how government administrators and principals of local schools respond to this inclusion–safety tension through harm-mitigating work, including reviewing students’ vaccination records, canceling extracurricular activities, and quarantining infected students (Pingali et al., 2019). In an extreme crisis, our model shows how fear of spreading an infectious disease can outweigh moral emotions of denying students access to education. For instance, in the wake of two recent measles outbreaks, custodians of schools in Clark County, Washington in the U.S. excluded unvaccinated students for several weeks (McRae, 2019). In addition, claims at local schools may pose threats of harm through violence (Lee, 2013), recognized as a problem of “persistence and pervasiveness throughout the history of education” (Cornell and Mayer, 2010: 7). School counselors, teachers, and administrators play roles as custodians through harm-mitigating work, including monitoring students’ communications and behavior and referring them for mental health support (Paolini, 2015). When fears escalate in the aftermath of a gun shooting (Newman, 2005), however, our model suggests that custodianship regarding the value of social inclusion may start to break down. Harm-avoiding proposals—such as arming teachers with guns or zero-tolerance policies that expel students for minor disciplinary infractions (Skiba and Knesting, 2001)—may emerge, disrupting the values of the public school as an accessible and safe place of social inclusion. As with Ebola in our study, disruptions from shootings and measles outbreaks represent extreme cases. More commonly, custodianship through resource-rationing, resource-enabling, and harm-mitigating work will maintain the local public school as a place of social inclusion.

Future research is needed to confirm the generalizability of our model depicting how custodianship processes unfold. Various other places of social inclusion established at institutional levels and instantiated at local levels warrant empirical investigation. Courts of law in democratic societies, for example, are charged with delivering impartial justice to all citizens (Parkinson, 2012), and local courthouses constitute the places where citizens come to access this legal justice (McPherson and Sauder, 2013). Public libraries and museums in towns and cities provide citizens with universal access to information, collective memory, and cultural heritage (Nelson and Irwin, 2015). The two defining characteristics of places of social inclusion could also apply to government sites offering employment services, public housing, community centers, social welfare agencies, and parks and recreation facilities (Altman and Zube, 1989). These places “protect the rights of user groups and are accessible to all groups” when citizens make claims at the local level for basic human needs (Carr et al., 1992: 19). Finally, places that are “physical sites of democratic performance” (Parkinson, 2012: 1), such as legislative assembly buildings, seem to fit the characteristics of a place of social inclusion when they remain open so that collective decision making is accessible and visible to citizens. While our main contribution is to shine light on places of social inclusion as a special type of institution, our study also contributes to the literatures on place and institutions, custodianship, and the role of emotions in institutional work.

### Place and Institutions

The institutions that we label places of social inclusion have been studied using other theoretical lenses. Scholars in institutional theory and the sociology of professions have explored how doctors, nurses, teachers, lawyers, librarians, museum curators, social workers, and other professionals maintain and change the logics, practices, values, identity, and status of professions within public emergency departments (Wright, Zammuto, and Liesch, 2017), public schools (Arum, 2000; Hallett, 2010), drug courts (McPherson and Sauder, 2013), public libraries (Nelson and Irwin, 2015), public museums (Blagoev, Felten, and Kahn, 2018), and the like. An implicit assumption is that these are places of professional work inside public-sector organizations. Our findings challenge this assumption because typifications of profession and organization are inadequate for capturing how societal-level values inhere in places at the local level. Framing geographic sites narrowly as places of professional work avoids attention to the higher level of social purpose that some of these places are mandated to fulfill in democratic society (Selznick, 1992). Viewing these places as public-sector organizations prioritizes questions associated with organizational goals and management processes (Kraatz, Ventresca, and Deng, 2010), overlooking citizens’ expectations and lived experience of them not as organizations but as local places to which they are entitled access. Conjoining profession with public organization disregards the tensions between social inclusion and local resources and safety that make places of social inclusion precarious.

Our conceptual insights into places of social inclusion move the literature forward by elucidating a puzzle that cuts across the literatures in sociology, public administration, and institutional theory. This puzzle asks “can a social purpose that is fundamentally experiential be institutionalized at all?” (Nilsson, 2015: 370) and leads to contradictions such as “how schools can at once be egalitarian institutions and agents of inequality” (Domina, Penner, and Penner, 2017: 311). In contrast to customary explanations anchored in professional, administrative, and organizational failure (Rowan, 1982; Raudenbush and Eschmann, 2015), our findings suggest these contradictions are related to the constitution of places of social inclusion as nested societal and local institutions. Whether an egalitarian institution at the societal level functions as an agent of equality or inequality at the local level depends on the volume and nature of claim-makers at a local place; it also depends on the subjective judgments of claim-responders with regard to tensions between universal access and the finite resources and safety of the local place. By revealing how the institutional dynamics of “claim making” and “responding to claims” are tailored to local places, our model gives researchers and policymakers a new piece of the puzzle of “experienced inequality” (Selznick, 1992: 383), which was obscured when places of social inclusion were viewed through other theoretical lenses.

Our identification of the concept of a place of social inclusion challenges researchers to consider the implications and potential scope conditions for established theories. Institutional complexity is likely to manifest differently when professionals work in these special places (Greenwood et al., 2011). Our findings intimate, for example, that some of the public defenders in McPherson and Sauder’s (2013) study of a drug court may have been more willing to stray from their home professional logic and strategically hijack other logics because they identified as custodians of a place of social justice and were engaged in custodianship to manage resource and safety tensions. Thus our model contributes place-based insights that may help to clarify and refine theories of how professionals function as institutional agents. In a different vein, our study offers a theoretical avenue for reconciling conflicting results about the “paradox of expertise” when new technology emerges in professional work (Lewis, 2000). Notably, librarians in public libraries ignored the Internet as predicted by the paradox of expertise (Nelson and Irwin, 2015), while curators in public museums embraced it (Blagoev, Felten, and Kahn, 2018). Museum curators linked “the emerging technology of the Internet and the principle of providing universal access to their collection” (Blagoev, Felten, and Kahn, 2018: 1773), implying they responded to the Internet as custodians of a place of social inclusion whereas librarians responded as professionals. Custodian identity for librarians became activated later, when free access to online information created safety threats in local libraries by exposing children to inappropriate or offensive material (Jaeger et al., 2012). The theoretical insights from our study hint that custodianship of universal access may moderate the paradox of expertise.

In contributing to these literatures, we recognize that processes associated with professions and public-sector organizations also occur in places of social inclusion. These were clearly present in our study of a public hospital emergency department. What our findings unmask is a distinctive type of custodian work associated with a place of social inclusion that complements existing accounts offered by scholars of professions and public administration. This raises an obvious question. If public emergency departments, for example, are both places of medicine for professionals administered by public-sector organizations *and* places of social inclusion for society, how can researchers separate the institutional work directed at these different institutions? Our findings show that doctors and nurses working in a public ED where all citizens could receive services distinguished their professional work from that performed in private ED settings where only patients able to pay received treatment. They explained that their professional work as doctors and nurses was similar in both settings, but that in a public ED they were also engaged in custodian work to manage value tensions associated with universal access. This custodianship of the place of social inclusion was extra work overlaid on top of the normal work of patient diagnosis, care, and treatment required in a place of acute medicine. Thus, in conceptualizing places of social inclusion, we do not theorize that all work carried out is custodian work. What differentiates custodianship is its activation through perceived value tensions between the societal-level value of social inclusion and the local place.

The defining characteristics of places of social inclusion mean that place is intrinsic to their institutional constitution and reproduction in a way that has been undertheorized by existing explanations of how institutions function (Lawrence and Dover, 2015). While prior studies have typically reduced place to a site where work directed at other institutions happens (Siebert, Wilson, and Hamilton, 2017), our study reconceptualizes places as institutions in their own right. Our model reveals that rather than being mere background context, place is constitutive of an institution itself and consequential for the custodian work required to maintain it. We thus deepen theorizing about inhabited institutions (Hallett and Ventresca, 2006) by revealing how people inhabit institutions through their interactions with, and within, local places that are invested with societal values.

### Contributions to Custodian Work

Our study also contributes to the literature by offering fresh insight into how custodian work maintains institutions. Previous research has indicated that custodians identify with an institution and are committed to upholding its values and standards (Selznick, 1957; Soares, 1997), which motivates them to invest effort in custodian work to protect the institution (Dacin, Dacin, and Kent, 2019). Our findings call into question an implicit assumption in much of the custodianship literature about how custodians think about and engage with the boundaries of an institution. Although researchers have not focused explicitly on modes of engagement, the findings of prior research suggest this can be an important aspect of custodianship. Studies have reported that custodians maintain and protect institutions by restricting to insiders the performance rituals at the Cambridge dining hall and Texas A&M University’s Aggie Bonfire (Dacin and Dacin, 2008; Dacin, Munir, and Tracey, 2010), closing off the outside world’s access to the Scottish Advocates Library (Siebert, Wilson, and Hamilton, 2017), and excluding experiences not authentic to the remembered past of Oregon’s TrackTown (Howard-Grenville, Metzger, and Meyer, 2013). As these examples highlight, studies tend to assume a singular mode of engagement in which custodians work to contain the boundaries of institutional participation by separating those who belong within an institution’s values, norms, identities, and practices from those who do not. Our model of custodianship challenges this implicit assumption of boundary containment. We reveal an alternative mode of engagement in which custodians may purposefully direct effort to keep an institution’s boundaries open rather than contained.

Our findings show how custodian work that engages with institutional boundaries as permeable entails balancing a set of value tensions not evident when the mode of engagement is containment. Resource demands activate a distinctive value tension for custodians when boundaries are permeable. On the one hand, there might not be enough resources for custodians to keep the institution open for everyone seeking participation; on the other hand, closing off the institution’s boundaries to some or all participants to conserve resources violates values and expectations of the custodian role. This value tension elicits moral emotions and motivates custodian work to ration and enable resources in ways that keep the institution open for everyone. In contrast, resource demands do not appear to activate value tensions of this nature when the mode of engagement for custodian work is boundary containment. Thus the processes of resource rationing and resource enabling we find in places of social inclusion are distinctive from other forms of custodian work because the mode of engagement is boundary permeability rather than containment.

By bringing attention to permeability and containment as different modes of engagement with institutional boundaries, our study propels inquiry into custodianship in new theoretical directions. Building on our novel insight that managing value tensions associated with resources and safety distinguishes custodianship that engages with boundaries as permeable, future research could examine how these value tensions play out in custodianship in other institutions and explore other potential value tensions. One possibility is to investigate the power of dominant groups, such as when political pressures and interest groups at local levels try to undermine custodianship that seeks social inclusion (Lawrence and Dover, 2015; Lawrence, 2017). While the empirical data in our study do not allow us to examine the effects of local power, future research is warranted to explore power and other potential sources of tension in custodianship directed at boundaries.

### Emotions and Institutional Work

Our study contributes to the emerging stream of literature on emotions and institutional work (Voronov and Vince, 2012; Jarvis, 2017). We extend prior research that shows moral emotions play a role in institutional work by illuminating when and how they can activate custodian work that protects an institution’s values. Moral emotions are feelings that are prosocial and motivate action tendencies for the interests of others (Haidt, 2003; Tangney, Stuewig, and Mashek, 2007). Previous studies have focused attention on how moral emotions—including empathy for others, pride in moral rightness, and shame at moral wrongs—motivate institutional work to create, maintain, and change institutions (Moisander, Hirsto, and Fahy, 2016; Fan and Zietsma, 2017; Toubiana and Zietsma, 2017; Wright, Zammuto, and Liesch, 2017). Adding to this growing line of research, our study highlights how moral emotions motivate custodian work when actors care deeply about the values of an institution and perceive tension between those values and their accomplishment in local places. Moreover, our findings reveal that embedded actors can feel emotional attachment to both institutions and local places. Thus we speculate that the intensity of moral emotions may be stronger when institutional work is targeted at maintaining institutions that are constituted as places, although future research is needed to explore this possibility.

We also contribute to the emotions literature by casting new light on the role that fear plays in institutions. Prior research has offered two broad explanations. First, an institution’s regulations, norms, and systemic power arouse fear among institutional actors, which disciplines them to conform (Marti and Fernandez, 2013; Gill and Burrow, 2018). Second, institutional actors may use fear as a motivational force to collectively create, maintain, and change institutions (Moisander, Hirsto, and Fahy, 2016; Wijaya and Heugens, 2018). Both explanations are rooted in a socialized understanding of fear as being experienced in the context of institutions (Voronov and Vince, 2012). In contrast, our study of Ebola in a public emergency department reveals the potential for an alternative understanding in which fear is less directly connected to a person’s socialization within an institution and operates at a more basic level. This conceptualization resonates with the psychology literature on basic emotions, in which fear is an immediate intuitive reaction to a stimulus that triggers human behavior for survival (Ekman, 1992, 1999). Custodians’ initial fears about the Ebola virus were basic emotions, and the immediate instinct was self-defense to avoid personal harm. This distinction between fear as a socialized and basic emotion is not trivial. Whereas prior research has shown that socialized fear is an important and relatively straightforward mechanism in institutional maintenance (Creed et al., 2014; Gill and Burrow, 2018), our study reveals that institutional processes are more complicated when institutional actors feel fear as a basic emotion.

Our findings suggest that the impact of fear as a basic emotion is moderated by other types of socialized emotions within institutions, most notably moral emotions. In our study, moral emotions that were endogenously embedded in the institution of a place of social inclusion—including empathy for the needs of institutional participants and embarrassment at the possibility of failing to uphold the institution’s values—rose up to play off against fear triggered exogenously as a basic emotion by the environmental jolt of the Ebola outbreak. Through their self-consciously evaluative nature, moral emotions helped to dampen fear to the degree that custodians were able to fight against their own basic instincts for self-preservation and engage in institutional work to maintain the institution. A comparable interplay of moral emotions and basic emotions appears to have normalized fears associated with custodian work in response to familiar infectious diseases and patient violence. Thus our model extends prior research on moral emotions by illuminating their critical role in moderating fear, which otherwise has the potential to derail institutional maintenance. We speculate that if a patient suffering from Ebola or behaving violently caused serious harm or death to others, basic emotions might overwhelm more reflective moral emotions and disrupt ordinary processes of institutional maintenance. We call for further research exploring the dynamics of fear and institutional disruption, which events associated with the COVID-19 pandemic suggest is an issue of vital global importance for places of social inclusion.

Interplay between basic and moral emotions in the unfolding processes of institutional work over time is a unique aspect of our study. Prior empirical studies have tended to ignore basic emotions, possibly because they seem à priori to be less theoretically salient to institutions than socialized emotions. The study of emotions in institutions has also been hampered by methodological difficulties of tracing institutional actors’ emotions in real time (Goodrick, Jarvis, and Reay, 2019). Our study benefitted methodologically when an unexpected environmental jolt (Meyer, 1982) during our fieldwork cast the basic emotion of fear into sharp relief. Thus it is hardly surprising that the relationships between basic and moral emotions and institutional work revealed in our study have been largely hidden until now. We conjecture that these relationships are generalizable to other institutions beyond places of social inclusion. We suspect, for example, that institutional work to create Canada’s first safe narcotics injection sites in Lawrence’s (2017) study and institutional work to disrupt organized crime in Sicily in Vaccaro and Palazzo’s (2015) study were both enabled by moral emotions controlling institutional actors’ basic emotions of fear. In addition to exploring the experience of fear in other types of institutions, we invite investigation of other basic emotions identified in the psychology literature, including anger, disgust, happiness, sadness, and surprise (Ekman, 1992, 1999). Future research into institutional work could explore when and how instinctive basic emotions work with and against higher-order reflective emotions that are more closely connected with actors’ socialization and embeddedness within institutions, such as moral anger and betrayal (Toubiana and Zietsma, 2017; Wijaya and Heugens, 2018), shame (Creed et. al., 2014), other-suffering empathy (Wright, Zammuto, and Liesch, 2017), and pride (Fan and Zietsma, 2017).

### Conclusion

Our study brings attention to places of social inclusion as important institutions of the democratic state that provide citizens with universal access to essential human services. We offer insight into how these institutions are maintained across nested societal and local levels through the purposeful work of custodians. We invite research exploring custodianship in diverse places of social inclusion, ranging from places with venerable histories of inclusion like public schools and museums to recently emerging places like sanctuary cities. Our findings about how such places endure are timely in light of the overwhelming fear and disruptions to places of social inclusion in the wake of the global coronavirus pandemic and given contested political discourse in the U.S. and Europe about what it means for nations, cities, and communities to be socially inclusive places. We encourage scholars to explore places of social inclusion and to expand the theorization and empirical investigation of place and institutions.

## Footnotes

1 The data analysis process was also informed by the lead author’s own experiences at the fieldsite emergency department when her daughter became ill. The implications of this collision between research and personal lifeworlds have been explored in Wright and Wright (2019).

2 To protect the confidentiality of study participants, we identify interview respondents by the following codes: D = senior doctor, R = resident, N = nurse, M = hospital manager. We do not include dates for fieldnote extracts to avoid the identification of individuals.
